# Profile of Neonates Admitted With Hypoxic‐Ischemic Encephalopathy and Early Neurodevelopment Outcomes: A Retrospective Cohort Study

**DOI:** 10.1111/jpc.70588

**Published:** 2026-09-13

**Authors:** Visothpong Chhoam, Nicole Ng, Abdul Razak, Vathana Sackett, Annie Bourke, Pramod Pharande, Rod W. Hunt, Lindsay Zhou, Atul Malhotra

**Affiliations:** ^1^ School of Clinical Sciences at Monash Health, Monash University Melbourne Australia; ^2^ Department of Paediatrics Monash University Melbourne Australia; ^3^ Monash Newborn, Monash Children's Hospital Melbourne Australia; ^4^ The Ritchie Centre, Hudson Institute of Medical Research Melbourne Australia; ^5^ Newborn Services, Joan Kirner Women's and Children's Hospital Melbourne Australia; ^6^ Allied Health Department Monash Children's Hospital Melbourne Australia; ^7^ Cerebral Palsy Alliance Sydney Australia

## Abstract

**Objective:**

To evaluate contemporary clinical profiles and early neurodevelopmental outcomes of neonates admitted with hypoxic–ischemic encephalopathy (HIE) in an Australian tertiary perinatal unit.

**Study Design:**

A retrospective cohort study (2019–2025) of neonates born ≥ 35 weeks' gestation admitted with a diagnosis of HIE. Early neurodevelopment outcomes were assessed at 3–4 months corrected age using standardised testing.

**Results:**

Of 114 neonates (mean gestation 39 weeks), 35/114 (30.7%) were determined to have mild and 79/114 (69.2%) moderate‐to‐severe HIE based on overall neonatal course. Comprehensive initial documentation on HIE staging was present in 72/114 (63.2%), with 42/114 (36.8%) requiring retrospective reconstruction of the modified Sarnat score. Therapeutic hypothermia was administered to 27/35 (77.1%) of the mild group, and to 71/79 (89.9%) of the moderate‐severe group. Mortality was 16/79 (20.3%) in the moderate‐to‐severe group and none in the mild group. Abnormal early neurodevelopmental outcomes were observed in 8/27 (29.6%) of the mild and 34/57 (59.6%) of the moderate‐to‐severe HIE cohort.

**Conclusion:**

Standardised documentation in HIE remains inconsistent in practice. Given the significant morbidity across all severity stages, this warrants closer scrutiny and reinforcement alongside structured long‐term neurodevelopmental surveillance for all affected neonates.

## 1 Introduction

Perinatal hypoxic‐ischemic encephalopathy (HIE) remains a critical cause of neonatal mortality and long‐term neurodevelopmental disability, with an incidence in high‐income countries of 1–3 per 1000 live births [1]. HIE is a result of hypoxia and ischemia to the newborn's brain, following a biphasic injury process: primary ischemia and secondary energy failure [2].

Therapeutic hypothermia (TH) is the standard of care during this crucial latent window. Cooling to 33°C and 34°C for 72 h [3] reduces metabolic demands, inflammation and oxidative injury [2]. Systematic reviews show TH reduces neonatal mortality by 25% [4] and significantly lowers rates of cerebral palsy, motor, cognitive and sensory impairments in survivors of moderate‐to‐severe HIE [5].

Guidelines from the American Academy of Paediatrics [3] and ANZCOR [6], recommend TH only for infants with evolving moderate‐to‐severe HIE identified within 6 h of birth, using standardised neurological examination [3, 4], followed by controlled rewarming at 0.5°C per hour. Despite this, mortality for HIE treated with TH has plateaued near 12.3% [7] and 30% of survivors experience major disability [8].

A critical diagnostic gap persists: infants with mild HIE are not uniformly benign neurodevelopmentally [9]. Mild HIE is associated with worse outcomes at 2 years versus controls [10], with 16% uncooled infants showing disability. This uncertainty has driven ‘therapeutic drift’, where clinicians cool infants outside strict criteria, including for infants at 34–35 weeks' gestation or those with mild HIE [11, 12, 13]. The COMET trial is currently underway evaluating TH safety and efficacy in mild HIE [14].

Contemporary care increasingly emphasises early neurodevelopmental assessment. Combining the Prechtl General Movements Assessment (GMA), Hammersmith Infant Neurological Examination (HINE) and early MRI enables identification of infants at high risk of cerebral palsy (CP) and developmental delay by 3–4 months corrected age [15, 16]. However, limited data describe detailed clinical profiles of neonates with HIE during admission and their association with early neurodevelopmental outcomes. Therefore, this study evaluated this association. Assessment at this early time point is important, as early neurodevelopmental findings at 3–4 months corrected age have been shown to correlate strongly with longer‐term neurodevelopmental outcomes [15].

## 2 Methods

### 2.1 Study Design and Setting

This retrospective cohort study was conducted at Monash Children's Hospital in Melbourne, Australia. Monash Children's Hospital supports over 10 000 births annually in the Monash Health network and functions as the largest tertiary neonatal service in Victoria.

Infants were identified using the local Australian and New Zealand Neonatal Network (ANZNN) database and data were collected to retrieve maternal, perinatal and postnatal variables from the participants' electronic medical records.

Inclusion criteria comprised neonates born ≥ 35 weeks' gestation between February 2019 and November 2025 with a HIE diagnosis. Exclusion criteria included neonates with postnatal cardiac arrest beyond 6 h of life or cases where severity could not be determined despite full record review.

### 2.2 Ethics Statement

Approved by Monash Health Human Research Ethics Committee as a quality assurance project (QA/81909/MonH‐2022‐207 997 (v1)).

### 2.3 HIE Diagnosis and Staging

Modified Sarnat score was used and a diagnosis of moderate‐to‐severe HIE was made when three or more domains fell within the abnormal range. HIE was staged at two time points: the initial diagnosis and the final (overall) diagnosis.

The initial HIE stage was based on neurological assessment within the first 6 h of life based on modified Sarnat scoring. Where formal scoring was absent, the initial stage was derived from recorded clinical examination notes during the first 6 h. The final diagnosis was guided by the ANZNN classification, incorporating MRI imaging, amplitude‐integrated electroencephalography (aEEG), clinical evolution of neurological status and discharge assessment. Any neonate with confirmed electrical or clinical seizures, whether prior to cooling or at any point during admission, was classified as at least moderate HIE. All primary analyses in this study were assessed according to the final overall HIE stage.

### 2.4 Neurodevelopmental Outcomes

Infants had early neurodevelopment assessments at 3–4 months of corrected age by a multidisciplinary team, consisting of a consultant neonatologist, physiotherapist and/or occupational therapist, who performed the GMA and the HINE [15, 17]. The HINE was scored on a scale of 0–78, with a score of less than 57 at 3–4 months of corrected age considered abnormal [17]. Additionally, the GMA was conducted via video analysis or direct observation. Results were categorised as normal fidgety, absent fidgety or abnormal fidgety [18]. Based on this, infants were classified as normal, developmental delay or early features of CP or at high risk of CP [15, 16, 19, 20]. Agreement between trained assessors performing GMA and HINE, which are independently validated screening tools, was not formally assessed in this study.

### 2.5 Statistical Analysis

Statistical Analyses were performed using Microsoft Excel (Microsoft Corporation, Redmond, WA, USA) and GraphPad Prism Version 10.0 (GraphPad Software, San Diego, CA, USA). The normality of numerical data was tested by the D'Agostino & Pearson test. The mean (standard deviation) was calculated for normally distributed data, while the median (interquartile range) was calculated for non‐normally distributed data. Post hoc comparisons between mild and moderate‐severe HIE groups were performed using Welch's *t*‐test for normally distributed continuous variables and Fisher's exact test (or chi‐square where expected cell counts permitted) for categorical variables. A *p*‐value of < 0.05 was considered statistically significant.

## 3 Results

### 3.1 Participants

One hundred and eighteen infants met the inclusion criteria and four were excluded (postnatal arrest occurring beyond 6 h (*n* = 2), HIE severity undetermined *n* = 2), and a final cohort of 114 neonates were included (Figure 1). Of these, 16/114 (13.9%) died in the neonatal period. Among the survivors, 6/98 were lost to follow‐up for early neurodevelopmental assessments, 4/98 (4%) did not meet criteria for early neurodevelopmental assessments and 4/98 (4%) were transferred to another hospital system for ongoing follow‐up care. 84/98 (85.7%) surviving neonates underwent early neurodevelopmental assessments, including 27/35 (77.1%) with mild HIE and 57/63 (90.5%) with moderate‐to‐severe HIE.

**FIGURE 1:**
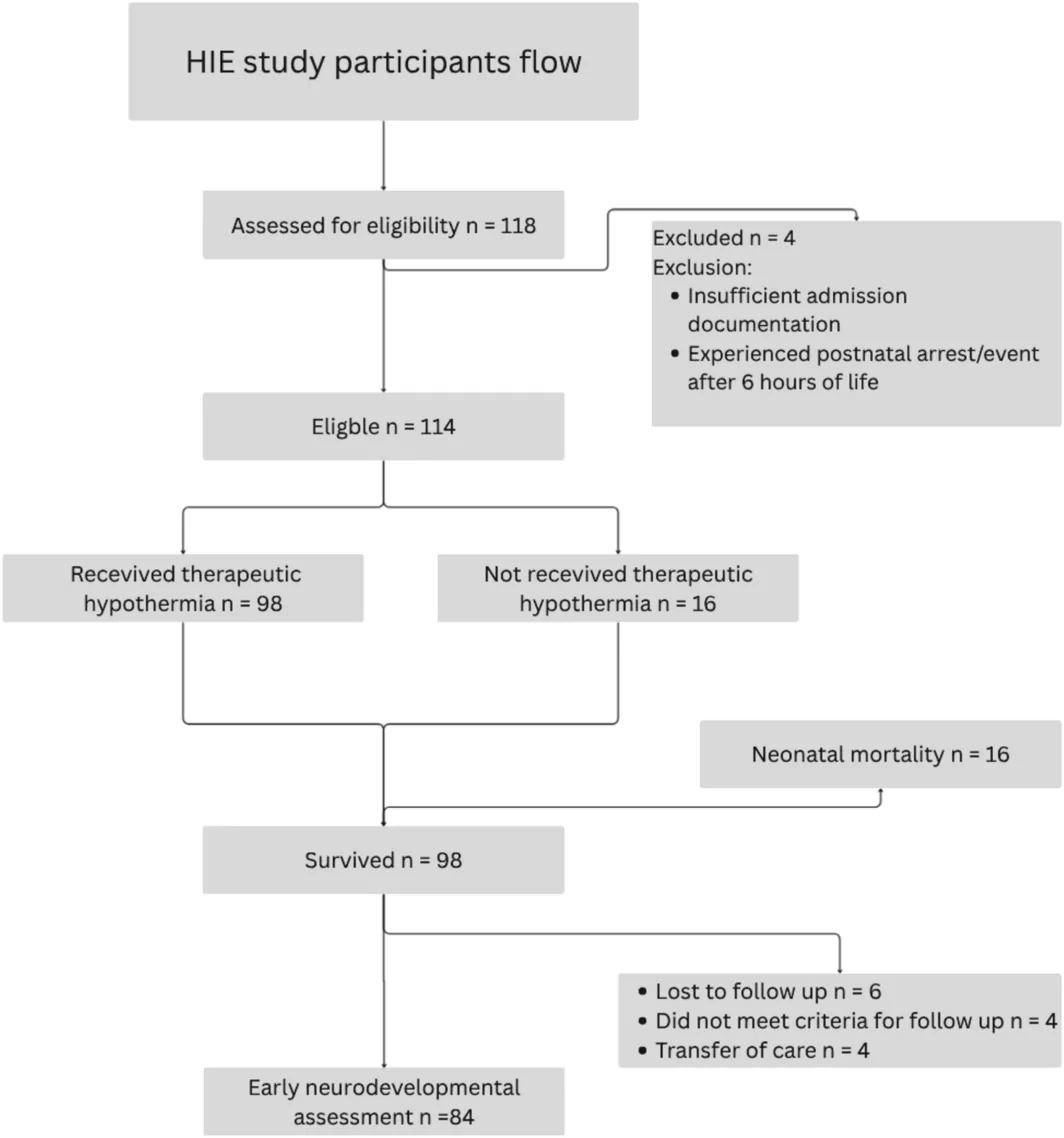
Participant flow.

With respect to data completeness, Apgar scores at 10 min were unavailable for 16/114 (13.9%) neonates. Initial blood gas pH values were missing for 6/114 neonates, and initial lactate levels were not recorded for 2/114 neonates. Brain magnetic resonance imaging (MRI) was not performed in 15/114 neonates, and aEEG was not conducted in 13/114 neonates.

### 3.2 Documentation

Of the 114 neonates included in the cohort, 72/114 (63.2%) had comprehensive documentation of the initial HIE diagnosis and stage, whereas the remaining 42/114 (36.8%) required retrospective reconstruction from clinical documentation. With respect to final HIE staging, 54/114 (47.4%) neonates had no documented HIE stage in their discharge summaries. Furthermore, no formal Sarnat score was recorded in 114 neonates [21].

### 3.3 Demographics

Of the 114 neonates, 35/114 (30.7%) were classified as having mild HIE and 79/114 (69.2%) as having moderate‐to‐severe HIE. Demographic, perinatal and maternal characteristics are presented in Table 1. Notably, 27/35 (77.1%) of the mild HIE group received therapeutic hypothermia, of whom 24/35 (68.6%) completed the full 72‐h protocol, compared with 69/79 (87.3%) in the moderate‐to‐severe group. Mortality was 16/79 (20.3%) in the moderate‐to‐severe group and 0/35 (0%) in the mild HIE group.

**TABLE 1:** Demographic, clinical, and inpatient outcomes of neonates admitted with hypoxic‐ischemic encephalopathy, stratified by final HIE severity.

		Mild HIE	Moderate‐severe HIE	*p*
Demographic	Gestation age (weeks), mean (SD)	38.9 (1.7)	39.0 (1.6)	0.77
	Birth weight (g), mean (SD)	3266 (612.2)	3288 (699)	0.87
	Male sex, *n* (%)	17/35 (48.6%)	53/79 (67.1%)	0.06
	Outborn, *n* (%)	23/35 (65.7%)	53/79 (67.1%)	0.89
	Caesarean, *n* (%)	17/35 (48.6%)	45/79 (57%)	0.41
Pregnancy complications	Pre‐Eclamptic Toxaemia, *n* (%)	2/35 (5.7%)	4/79 (5.1%)	0.99
	Gestation diabetes mellitus, *n* (%)	5/35 (14.3%)	15/79 (19%)	0.54
Intrapartum complications	Shoulder dystocia, *n* (%)	4/35 (11.4%)	7/79 (8.9%)	0.67
	Placental abruption, *n* (%)	3/35 (8.6%)	14/79 (17.7%)	0.21
	Cord prolapse, *n* (%)	4/35 (11.4%)	4/79 (5.1%)	0.22
	Uterine rupture, *n* (%)	1/35 (2.9%)	5/79 (6.3%)	0.44
Resuscitation	1 min APGAR, median (IQR)	1 (1, 3)	2 (1, 3)	0.10
	5 min APGAR, median (IQR)	4 (3, 6)	4 (2, 5)	0.27
	10 min APGAR, median (IQR)	6 (4, 7)	5 (3, 6)	0.03
	Intermittent positive pressure ventilation, *n* (%)	33/35 (94.3%)	71/79 (89.9%)	0.44
	Intubation at delivery, *n* (%)	12/35 (34.2%)	44/79 (55.7%)	0.03
	Chest compressions, *n* (%)	14/35 (40%)	36/79 (45.6%)	0.58
	Adrenaline, *n* (%)	3/35 (8.6%)	17/79 (21.5%)	0.09
	Continued resuscitation > 10 min at birth, *n* (%)	18/35 (51.4%)	58/79 (73.4%)	0.02
	Initial pH, mean (SD)	7.0 (0.1)	7.0 (0.2)	1
	Initial base excess, mean (SD)	−14.3 (3.6)	−16.3 (6.1)	0.03
	Initial lactate, mean (SD)	11.9 (3.8)	13.7 (5.0)	0.03
Inpatient outcomes	Any duration of therapeutic hypothermia, *n* (%)	27/35 (77.1%)	71/79 (89.9%)	0.07
	72 h of therapeutic hypothermia, *n* (%)	24/35 (68.6%)	69/79 (87.3%)	0.01
	Confirmed seizures, *n* (%)	0/35 (0%)	65/79 (82.3%)	< 0.001
	No anti‐epileptic drugs required, *n* (%)	34/35 (97.1%)	15/79 (20.3%)	< 0.001
	Monotherapy anti‐epileptic drugs, *n* (%)	1/35 (2.9%)	32/79 (40.5%)	< 0.001
	> 1 Anti‐epileptic drugs, *n* (%)	0/35 (0%)	32/79 (39.2%)	< 0.001
	Intubated during stay, *n* (%)	17/35 (48.6%)	66/79 (83.5%)	< 0.001
	Abnormal aEEG/EEG, *n* (%)	3/28 (10.7%)	50/73 (68.5%)	< 0.001
	MRI evidence of HIE, *n* (%)	7/25 (28%)	38/74 (51.4%)	0.04
	Length of stay (days), median (IQR)	9 (7, 13)	13.5 (9, 19.75)	0.0002
	Died, *n* (%)	0/35 (0%)	16/79 (20.3%)	0.004
	Lost to follow up, *n* (%)	4/35 (11.4%)	6/63 (9.5%)	0.77

### 3.4 Early Neurodevelopment Outcomes (Table 2)

**TABLE 2:** Early neurodevelopmental outcomes at 3–4 months corrected age among survivors of hypoxic‐ischemic encephalopathy, stratified by final HIE severity.

		Mild HIE	Moderate‐ severe HIE	*p*
Early neurodevelopmental outcomes	Did not meet early neurodevelopment clinic referral criteria, *n*/*N* (%)	4/35 (11.4%)	0/63 (0%)	0.014
	Attended early neurodevelopment clinic, n/N (%)	27/35 (77.1%)	57/63 (90.5%)	0.12
	Corrected gestational age at appointment (weeks), median (IQR)	13.4 (13, 14.1)	13.4 (12.8, 14.3)	1
	Absent fidgety GMAs, *n*/*N* (%)	1/27 (3.7%)	20/57 (35.1%)	0.001
	HINE score, mean (SD)	58.3 (9.6)	51.9 (10.8)	0.008
	HINE score < 57, *n*/*N* (%)	9/27 (33.3%)	34/57 (59.6%)	0.03
	Normal development, *n*/*N* (%)	19/27 (70.4%)	23/57 (40.4%)	0.01
	Developmental delay, *n*/*N* (%)	5/27 (18.5%)	14/57 (24.6%)	0.59
	Cerebral Palsy/high risk of Cerebral Palsy, *n*/*N* (%)	3/27 (11.1%)	20/57 (35.1%)	0.03

Notably, 4/35 (11.4%) of the mild group did not meet referral criteria for neurodevelopmental assessment, compared to none in the moderate‐to‐severe group. Absent fidgety movements were identified in 1/27 (3.7%) in the mild group and 20/57 (35.1%) of the moderate‐to‐severe group. The mean (SD) HINE score was 58.3 (9.6) for mild HIE and 51.9 (10.8) for moderate‐to‐severe HIE. Overall, 19/27 (70.4%) of the mild cohort and 23/57 (40.4%) of the moderate‐to‐severe cohort had normal development. Developmental delay occurred in 5/27 (18.5%) and 14/57 (24.6%) of the groups, while high risk of cerebral palsy was identified in 3/27 (11.1%) and 20/57 (35.1%) of the mild and moderate‐to‐severe groups, respectively.

## 4 Discussion

This study evaluated the clinical profiles and early neurodevelopmental outcomes of a contemporary group of infants with HIE. A significant finding was the presence of documentation gaps, with one third of the cohort requiring retrospective determination of initial HIE staging. Additionally, 68.6% of the mild HIE cohort received therapeutic hypothermia, reflecting ‘therapeutic drift’. Despite this, 8/27 (29.6%) of infants had abnormal early neurodevelopmental outcomes. These findings highlight significant morbidity across all HIE severity stages and underscore the need for standardised neurological documentation and long‐term neurodevelopmental follow‐up for all affected neonates.

### 4.1 Documentation

A critical observation in this study was the lack of formal neurological documentation. While 114 neonates were identified with HIE, comprehensive initial staging was documented for only 72/114 (63.2%) of the population, necessitating a retrospective reconstruction of HIE stages for the remaining 42/114 (36.8%). Similar findings were noted by Venter et al. [22], where electronic discharge summaries frequently lacked the granular detail required for precise HIE staging.

To address these diagnostic inconsistencies, the implementation of a standardised screening framework, such as the Neonatal Encephalopathy Trigger Tool, may be essential. Peebles et al. [23] demonstrated that utilising objective biochemical and perinatal markers to ‘trigger’ an immediate clinical assessment significantly improves the identification rate of eligible neonates and reduces the time to initiate therapeutic hypothermia. However, the success of such tools is heavily dependent on clinical staff. Evidence indicates that structured education can significantly improve diagnostic competence; for instance, a quality improvement initiative [21] found that formal Sarnat training increased the percentage of staff correctly scoring clinical scenarios ‘from 12% to 71%’. Furthermore, ongoing multidisciplinary simulation training is vital for maintaining reliability in assessing subjective components such as muscle tone and the Moro reflex, supporting consistent application of HIE staging criteria across clinical teams [24]. Findings from this study have prompted a local quality improvement project at our institution, yet in development phase, incorporating a standardised, prompted HIE documentation proforma and structured staff education, with planned re‐audit to assess improvement in documentation completeness over time.

### 4.2 Therapeutic Drift

Another critical point is the high rate of TH observed in the mild HIE cohort and its relationship with neurodevelopmental outcomes. In this study, 27/35 (77.1%) of infants with mild HIE received any duration of TH, of whom 24/35 (68.6%) completed the full 72‐hour protocol, reflecting a clinical trend of ‘therapeutic drift’ whereby clinicians initiate neuroprotection in neonates who exhibit significant initial distress but may not strictly meet criteria for moderate‐to‐severe encephalopathy. The discrepancy between initiation and completion rates suggests that cooling was discontinued early in a subset of infants, potentially reflecting clinical reassessment of eligibility or improvement in neurological status during the cooling period. While TH is the established standard of care for reducing mortality and major disability in moderate to severe HIE [4, 25], its application in the mild HIE group remains controversial due to the lack of proven efficacy and the potential for physiologic harm. Research indicates that TH is associated with significant systemic complications, including sinus bradycardia, hypotension and thrombocytopenia, which may unnecessarily burden neonates who would have otherwise recovered spontaneously [4].

Despite these risks, the high incidence of suboptimal outcomes in the mild cohort suggests that mild HIE remains an evolving and high‐risk diagnosis. The limitations of current clinical staging in accurately predicting outcomes in mildly affected infants may itself be driving therapeutic drift. Clinicians who are uncertain of the true severity may elect to intervene with TH as a precautionary measure. With the widespread of therapeutic drift [12], the COMET trial [14] and other long‐term neurodevelopmental assessments are needed to validate the utility of TH in mild HIE. These findings underscore that HIE is a dynamic clinical state in which the potential neuroprotective benefits of therapeutic hypothermia must be carefully weighed against the risks of overtreatment, particularly in mild presentations.

### 4.3 Early Neurodevelopmental Outcomes

Most notably, the data revealed that infants categorised as having mild HIE at birth were not without risk for significant morbidity. In the mild cohort, only 19/27 (70.4%) of infants were classified as having normal development, while 5/27 (18.5%) exhibited developmental delay and 3/27 (11.1%) were identified as having cerebral palsy or being at high risk for CP. This combined 8/27 (29.6%) rate of suboptimal outcomes in the mild group highlights a critical clinical concern, as these infants have traditionally been viewed as having a universally benign trajectory, a finding that mirrors recent longitudinal studies suggesting that ‘mild’ HIE may harbour significant neurodevelopmental risks that are often underestimated at birth [10, 26].

The qualitative assessment of motor patterns via GMAs provided a clearer distinction between the two cohorts than the numerical HINE scores alone, with absent fidgety movements recorded in 20/57 (35.1%) of the moderate/severe group compared to 1/27 (3.7%) in the mild group. This supports the utility of GMAs as a highly sensitive predictor for severe motor impairment such as cerebral palsy, even when standardised scores like the HINE show variability [18]. Nevertheless, the high incidence of developmental concerns in the mild HIE group is particularly striking given that these infants are frequently less likely to receive the same intensity of neurodevelopmental surveillance as their moderate‐to‐severe counterparts. Notably, 4/35 (11.4%) of mild infants did not meet referral criteria for early neurodevelopmental assessment, compared to none of the moderate‐to‐severe group. These results underscore the necessity for standardised, long‐term follow‐up for all infants with HIE, ensuring that those in the ‘mild’ category are not lost to early intervention services despite their seemingly lower risk at birth. Given the clinical significance of an 11.1% rate of CP or high risk of CP identified in the mild HIE group, longer‐term follow‐up of this cohort is planned to confirm these early findings.

## 5 Limitations

This study's retrospective design introduced inherent challenges with data completeness and classification. Specifically, 43/114 (37.7%) of the cohort lacked formal neurological staging at birth, necessitating a reconstruction of HIE severity from clinical notes. This non‐uniform methodology introduces potential misclassification bias and heterogeneity; however, the impact is likely mitigated as primary analyses were stratified by final HIE diagnoses guided by the standardised ANZNN framework.

Selection and referral biases also impact the findings. While survival was high, 11.4% of mild HIE infants were not referred for neurodevelopmental assessments, suggesting that infants perceived as ‘low risk’ may have missed surveillance. Furthermore, because 77.1% of the mild cohort received therapeutic hypothermia, it is difficult to determine if favourable outcomes reflect the natural history of the disease or the neuroprotective effects of the intervention.

Furthermore, early neurodevelopmental assessment may not fully capture long‐term motor or cognitive outcomes, as evidence suggests that while such assessments can reliably identify cerebral palsy, their accuracy in detecting cognitive impairment and overall neurodevelopmental impairment remains limited, necessitating longer‐term follow‐up for a more complete picture [15].

Finally, the confounding created by ‘therapeutic drift’ must be acknowledged. Because 27/35 (77.1%) of the mild HIE group received therapeutic hypothermia, it is difficult to determine if the 70.4% rate of normal development reflects the natural history of mild HIE or the neuroprotective effects of the intervention itself.

## 6 Conclusions

This study demonstrates that HIE carries significant morbidity across all severity stages, with abnormal early neurodevelopmental outcomes observed in 29.6% of mild and 59.6% of moderate‐to‐severe survivors, challenging the assumption that mild HIE follows a benign trajectory. These findings highlight critical gaps in neurological documentation and underscore the need for standardised assessment and mandatory long‐term neurodevelopmental surveillance for all neonates with HIE.

## Funding

This research received no specific grant from any funding agency in the public, commercial, or not‐for‐profit sectors. Prof Malhotra's research is supported by NHMRC, Medical Research Future Fund, NSCFA, Cerebral Palsy Alliance, Monash Health Foundation & Lions Cord Blood Foundation.

## Ethics Statement

Ethics approval was granted by the Monash Health Human Research Ethics Committee as a quality assurance project (Ethics Reference: QA/81909/MonH‐2022‐207 997 (v1)). The study was performed in accordance with the Declaration of Helsinki.

## Conflicts of Interest

The authors declare no conflicts of interest.

## Data Availability

The data that support the findings of this study are available on request from the corresponding author. The data are not publicly available due to privacy or ethical restrictions.
